# Antibody to mCLCA3 Suppresses Symptoms in a Mouse Model of Asthma

**DOI:** 10.1371/journal.pone.0082367

**Published:** 2013-12-09

**Authors:** Liqiang Song, Dapeng Liu, Changgui Wu, Shouzhen Wu, Junlan Yang, Fangping Ren, Yan Li

**Affiliations:** 1 Department of Pulmonary and Critical Care Medicine, Xijing Hospital, Fourth Military Medical University, Xi’an, China; 2 Department of Pharmacy, The Fourth Military Medical University, Xi’an, China; 3 Department of Cardiovascular Medicine, Xijing Hospital, Fourth Military Medical University, Xi’an, China; University of Houston, United States of America

## Abstract

**Background:**

Asthma is a complex and heterogeneous chronic inflammatory disorder that is associated with mucous cell metaplasia and mucus hypersecretion. Functional genomic analysis indicates that mucous cell metaplasia and mucus hypersecretion depend on members of the calcium-activated chloride channel (CLCA) gene family. It has been reported that the inhibition of CLCAs could relieve the symptoms of asthma. Thus, the mCLCA3 antibody may be a promising strategy to treat allergic diseases such as asthma.

**Methods:**

We constructed asthmatic mouse models of OVA-induced chronic airway inflammatory disorder to study the function of the mCLCA3 antibody. Airway inflammation was measured by HE staining; goblet cell hyperplasia and mucus hypersecretion were detected by PAS staining; muc5ac, IL-13, IFN-γ levels in bronchoalveolar lavage fluid (BALF) were examined by ELISA; Goblet cell apoptosis was measured by TUNEL assay and alcian blue staining; mCLCA3, Bcl-2 and Bax expression were detected by RT-PCR, Western blotting and immunohistochemical analysis.

**Results:**

In our study, mice treated with mCLCA3 antibody developed fewer pathological changes compared with control mice and asthmatic mice, including a remarkable reduction in airway inflammation, the number of goblet cells and mCLCA3 expression in lung tissue. The levels of muc5ac and IL-13 were significantly reduced in BALF. We also found that the rate of goblet cell apoptosis was increased after treatment with mCLCA3 antibody, which was accompanied by an increase in Bax levels and a decrease in Bcl-2 expression in goblet cells.

**Conclusions:**

Taken together, our results indicate that mCLCA3 antibody may have the potential as an effective pharmacotherapy for asthma.

## Introduction

Allergic asthma is increasingly regarded as a complex and heterogeneous chronic inflammatory disorder, involving a complex interplay between both environmental and genetic factors, and is becoming increasingly widespread worldwide, especially in developed countries [1]. Although allergic asthma is a complex disease, studies in patients and animal models have shown that reversible airflow obstruction including goblet cell hyperplasia, airway mucus hypersecretion and airway inflammation (including eosinophil infiltration) are the main hallmarks of the disease. 

In patients with bronchial asthma, activated eosinophils, mast cells and basophils release a variety of cytokines to promote the differentiation of Th cells into Th2 cells [2]. Th2 cells can secrete IL-4, IL-5, IL-10 and IL-13, which may result in mucus hypersecretion [3]. Moreover, these Th2 cytokines may be directly linked to the overexpression of CLCA in the asthmatic patient and asthmatic mouse models [4]. A previous study showed that the third murine CLCA homologue, mCLCA3, has been identified in goblet cells [5]. Goblet cell hyperplasia appears to be directly linked to CLCA over-expression in asthmatic mouse models. There is some evidence that the suppression of mCLCA3 inhibits goblet cell hyperplasia, whilst overexpression increases goblet cell number in mice [6]. The decrease in goblet cell number may be associated with apoptosis. Cell apoptosis contributes to the chronicity of an inflammatory process and could regulate inflammatory cell survival [7]. Apoptosis is controlled by suppressing or inducing genes such as *Bcl-2* and *Bax*. 

Calcium-activated chloride channels (CLCAs) are preferentially expressed on the secretory epithelium. Ten members of this family, derived from four mammalian species, have been identified and cloned: the four human homologues hCLCA1, hCLCA2, hCLCA3 and hCLCA4, the three murine homologues mCLCA1, mCLCA2 and mCLCA3, the bovine bCLCA1, the bovine lung endothelial cell adhesion molecule-1 bCLCA2, and the porcine pCLCA1 [8,9]. Several studies have implicated members of the CLCA family in cell cycle control, cell–cell adhesion, apoptosis, metastasis and tumorigenesis [10]. An increasing body of evidence suggests that members of the CLCA family play an important role in diseases with epithelial secretory dysfunctions [11]. The relationship between the CLCA family and asthma is the most commonly investigated aspect in CLCA studies. Functional studies combined with gene expression analysis indicate that mCLCA3 and hCLCA1 are associated with the development of mucous cell metaplasia in asthmatic mice [10]. Several studies on human tissue have previously demonstrated that hCLCA1 mRNA levels and protein expression are significantly increased in the airway epithelium of asthmatic patients [12,13]. Overexpression of mCLCA3 has previously been linked to goblet cell metaplasia and mucin overproduction in both *in vitro* and *in vivo* model systems [14]. Our previous study suggested that mCLCA3 plays a pivotal role in mucous overproduction by bronchial goblet cells and that an hCLCA1 DNA vaccine prevented mucus hypersecretion and related pathological changes in a murine asthma model through the induction of anti-mCLCA3 antibodies [15]. Therefore, CLCA proteins can serve as useful biomarkers as well as significant therapeutic targets for the diagnosis and treatment of patients with chronic inflammatory airway disease [10]. In this article, we used the asthmatic mouse models of OVA-induced chronic airway inflammatory disorder to study the function of the mCLCA3 antibody. We show that the mCLCA3 antibody can inhibit goblet cell hyperplasia and airway mucus hypersecretion in asthmatic mice. 

## Materials and Methods

### Antibodies

Rabbit anti- mouse mCLCA3 polyclonal antibody (ab46512, IgG, 1/8000 used for immunohistochemical analysis and 1/1000 used for western blotting[16,17]; the antibody reacts with mouse, but does not react with human), rabbit anti- mouse Bax polyclonal antibody (ab7977, IgG, 1/100 used for immunohistochemical analysis and 1/1000 used for western blotting[18]; the antibody reacts specifically with Bax and reacts with mouse, rat and human), rabbit anti- mouse Bcl2 polyclonal antibody (ab7973, IgG, 1/1000 used for immunohistochemical analysis and western blotting[18]) and rabbit anti- mouse β-actin polyclonal antibody (ab15263, IgG, 1/200 used for immunohistochemical and 1/3000 used for western blotting) were purchased from Abcam (Cambridge, MA). 

### Mice and sensitization

Female BALB/c mice aged 6–8 weeks (19~25 g) were obtained from the Experimental Animal Centre of the Fourth Military University, Shaanxi Province, China. Experiments were conducted under a protocol approved by the Institutional Animal Care and Use Committee of the Fourth Military University. OVA sensitization of BALB/c mice was performed as described previously with minor modifications [19]. Briefly, the BALB/c mice were sensitized by i.p. injections of 1 μg of OVA (Sigma, Saint Louis, MO) and 100 μg of Al(OH)_3_ suspended in 0.5 ml saline on days 0 and 7. On days 14–20, the BALB/c mice were challenged with 1% OVA aerosol for 5 h each day to construct an asthmatic mouse model and an antibody intervention asthmatic mouse model. On days 17–20, 50 μl mCLCA3 antibodies was dropped into the nasal passages of OVA-challenged mice to construct the antibody intervention asthmatic mouse model 30 min before OVA challenge. An equal quantity of normal saline was administered to asthmatic and control mice. Control mice were sensitized to phosphate-buffered saline (PBS) and challenged with PBS aerosols (PBS-mice). 

### BALF collection

BALF and serum were collected 24 h after the last OVA challenge, as described in the literature with minor modifications [19]. Briefly, mice were anaesthetized using 3% pentobarbital sodium. Mice were sacrificed after anesthesia. Tracheotomy was performed, and a cannula was inserted into the trachea. Half a milliliter of D-Hank′s (8.0 g NaCl, 0.4 g KCl, 0.12 g Na_2_HPO_4_·12H_2_O, 0.06 g KH_2_PO_4_, 1.0 g glucose, 2 mg 1% phenol red in 1000 ml double distilled water, pH 7.3~7.6) was placed in the trachea, and BALF was collected; the process was repeated three times. BALF was then centrifuged at 1500 RPM for 10 min at 4°C in a Beckman model TJ6 table-top centrifuge to obtain the supernatant, which was stored at -20°C before examination. 

### Histological examination

Histological examination was performed using HE staining according to the method described previously with minor modifications [20,21]. Briefly, mice were euthanized 24 h after the last OVA challenge or treatment with PBS aerosols. Lungs were removed and inflation-fixed through the trachea with 3% paraformaldehyde-PBS, washed with cold PBS, processed, embedded in paraffin blocks, and serially sectioned into 6-μm sections for histological analysis. The sections were deparaffinized and hydrated, and then stained with hematoxylin and eosin (H&E), or periodic acid-Schiff (PAS). Inflammation of the lungs was assessed by histological analysis of H&E-stained lung sections. Goblet cell hyperplasia of the airway epithelium was examined by histological analysis of PAS-stained sections. Quantification of goblet cells was performed by counting 500 epithelial cells, and then determining the percentage of PAS-stained epithelial cells in at least five airway cross-sections per slide. The experiment was repeated at least three times and five animals were included in each group.

### ELISA

Murine muc5ac and cytokines in BALF were measured using ELISA according to the manufacturers’ recommendations. The muc5ac ELISA was purchased from KeYingMei Technology Co. Ltd (Beijing, China). IL-13 and IFN-γ ELISA were performed using the Quantikine Murine IL-13 Kit and IFN-γ kit (R&D Systems, Minneapolis, MN).

### Real-time reverse transcription-PCR (RT-PCR)

RT-PCR estimation of messenger RNA (mRNA) levels was performed according to the manufacturer’s protocol. Total RNA was isolated from right lung tissues using Trizol reagent (Invitrogen, Carlsbad, CA). RT-PCR was performed by reverse transcription using 2 μg of total RNA plus a High-Capacity RNA-to-cDNA Kit and the appropriate primers. Primer sequences, PCR cycles and conditions were as follows: muc5ac: Sense 5’- AATGGCGAGTCTGTGCAGGA-3’, Antisense 5’- CACCAGGTGTGGCATTGT- GA-3’; mCLCA3, Sense 5’- AATGATGAGCCCTACACCGAACA-3’, Antisense 5’- AGTGAGCCCACTCATGGACAAAG-3’; Bax: Sense 5’- CAGGATGCGTCCACC- AAGAA-3’, Antisense 5’- CGTGTCCACGTCAGCAATCA; Bcl-2: Sense 5’- TGAAGCGGTCCGGTGGATA-3’, Antisense 5’- CAGCATTTGCAGAAGTCCTGTGA-3’; β-actin Sense 5'- CATCCGTAAAGAC- CTCTATGCCAAC -3’, Antisense, 5’- ATGGAGCCACCGATCCACA-3’. These primers were all synthesized by Takara Co., Ltd. (Tokyo, Japan). The cycling conditions were polymerase activation for 15 s at 95°C; 40 cycles of amplification at 95 °C, 5 s, 60°C, 20 s and 65°C,14 s. A cDNA fragment of β-actin was amplified as the control. 

### Western blot analysis

The total protein of lung tissue was extracted using RIPA buffer (Beyotime, Nantong, China) according to the manufacturer’s instructions, and the protein concentration was determined by BCA protein assay kit. Protein concentration was adjusted, and 30 mg of each sample was separated with 15% SDS-PAGE electrophoresis and transferred to a nitrocellulose membrane (Millipore). Then, membranes were blocked with 5% nonfat skim milk at 4°C overnight, and then blotted with primary antibodies at a working dilution of 1: 8000 at 4°C overnight. They were subsequently incubated with a solution of anti-rabbit IgG HRP-conjugated antibody (1:5000; Santa Cruz Biotechnologies) for 1 h at room temperature. Finally, the blots were visualized using enhanced chemiluminescence reagents. All experiments included five mice per group and were repeated at least three times.

### Immunohistochemistry

Immunohistochemistry studies were performed as previously described with minor modifications [19]. Briefly, mice were euthanized 24 h after the last PBS aerosol or OVA challenge. Lungs were removed and cut into 6-μm sections for immunohistochemistry analysis. The sections were deparaffinized with xylene and rehydrated in ethanol. Sections were blocked with 10% bovine serum for 30 min in a humidified chamber. Following this, sections were subsequently incubated with antibodies overnight at 4°C and visualized using confocal microscopy (Olympus Fluoview 500, Tokyo, Japan) with an excitation wavelength of 488 nm and emission range of 510–550 nm. Quantification of mCLCA3, Bax and BCl-2 was performed by counting the continuous 500 epithelial cells, and then determining the percentage of positive goblet cells in at least five airway cross-sections per slide. The experiments were repeated at least three times. All experiments included five mice per group.

### TUNEL and AB staining

TUNEL assay was performed using a commercially available kit according to the manufacturer's instructions (Roche, Nutley, NJ) and the method described previously [22]. Lungs were removed from mice and cut into 6-μm sections 24 h after the last PBS aerosol or OVA challenge. Following deparaffinization and rehydration, the sections were incubated with TdT enzyme and 11-digoxigenin dUTP at 37°C for 4 hours. After quenching the reaction, sections were then incubated with anti-digoxigenin FITC-conjugated antibody for 60 min at room temperature. After completion of the TUNEL process, the sections were then stained with alcian blue (AB) for 30 min. The cell nucleus was stained with hematoxylin. Yellowish-brown and brown staining was obtained with diaminobenzidine and hematoxylin (DAB, 1:50) and blue staining with AB. An equal volume of distilled water was substituted for the TdT enzyme as a negative control and sections were exposed to DNAase as a positive control. Images were acquired under a light microscope. The numbers of TUNEL- and AB-positive cells in the epithelia goblet cells were counted.

### Statistical analysis

Statistical analyses were performed using the SPSS17.0 software package. All data are presented as mean ± standard error of the mean (S.E.M.). Comparisons between groups were performed by analysis of variance (ANOVA) followed by LSD t-test. Differences were considered statistically significant at p<0.05. The correlation index was analyzed by Pearson's correlation coefficient analysis; P<0.05 was considered statistically significant.

## Results

### Effect of mCLCA3 antibody on OVA-induced airway inflammation

**Figure 1:**
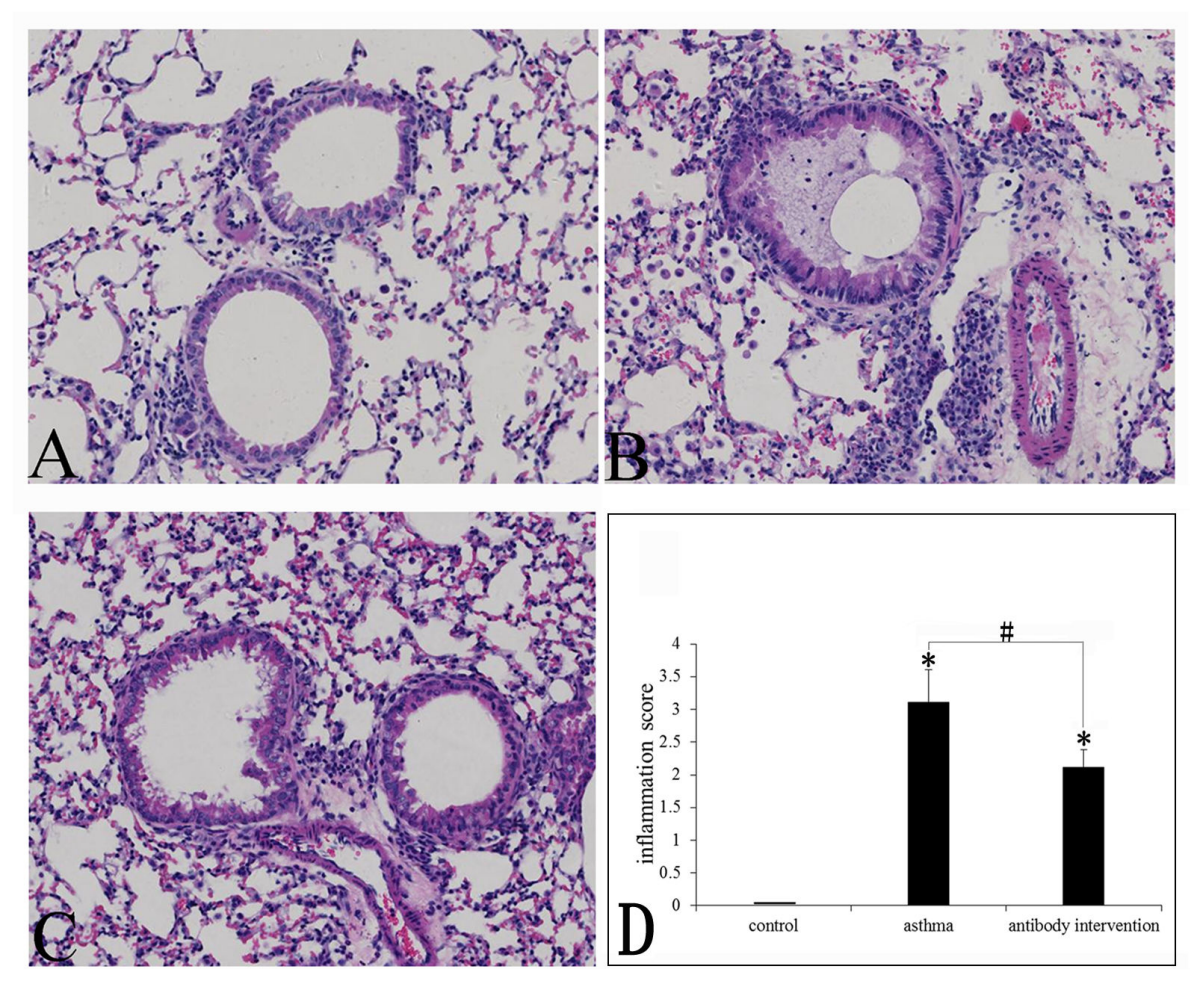
Effects of mCLCA3 antibody on OVA-induced airway inflammation. Histological examination of lung tissue by HE staining. (A) Control group; (B) Asthma group and (C) mCLCA3 antibody intervention group (magnification x400). (D) Quantitative analyses of airway inflammation in lung sections. Data are shown as mean ± SEM, n = 5. (*P<0.05, compared with the control group; ^#^P<0.05, compared with the asthma group).

Lung tissue was collected 24 h after the last OVA or PBS aerosol challenge. OVA challenge-induced airway inflammation was detected by HE staining. The results showed that in comparison with the control saline challenge, there was a significant increase in cell infiltration in the peribronchiolar and perivascular connective tissues in the OVA challenge group, including lymphocyte, monocyte and polymorphonuclear cells. The majority of the infiltrated inflammatory cells were eosinophils. Meanwhile, increased tracheal epithelium exuviation, airway wall thickness, luminal stenosis, and mucus plugs were found in the OVA-challenged mice. After treatment with mCLCA3 antibody, there was no significant difference in inflammatory cell infiltration; however, the antibody intervention asthmatic mice showed a marked improvement in epithelium exuviation, airway wall thickness, luminal stenosis, and mucus plug formation(Figure 1).

### Effect of mCLCA3 antibody on muc5ac and cytokine levels in BALF

**Figure 2:**
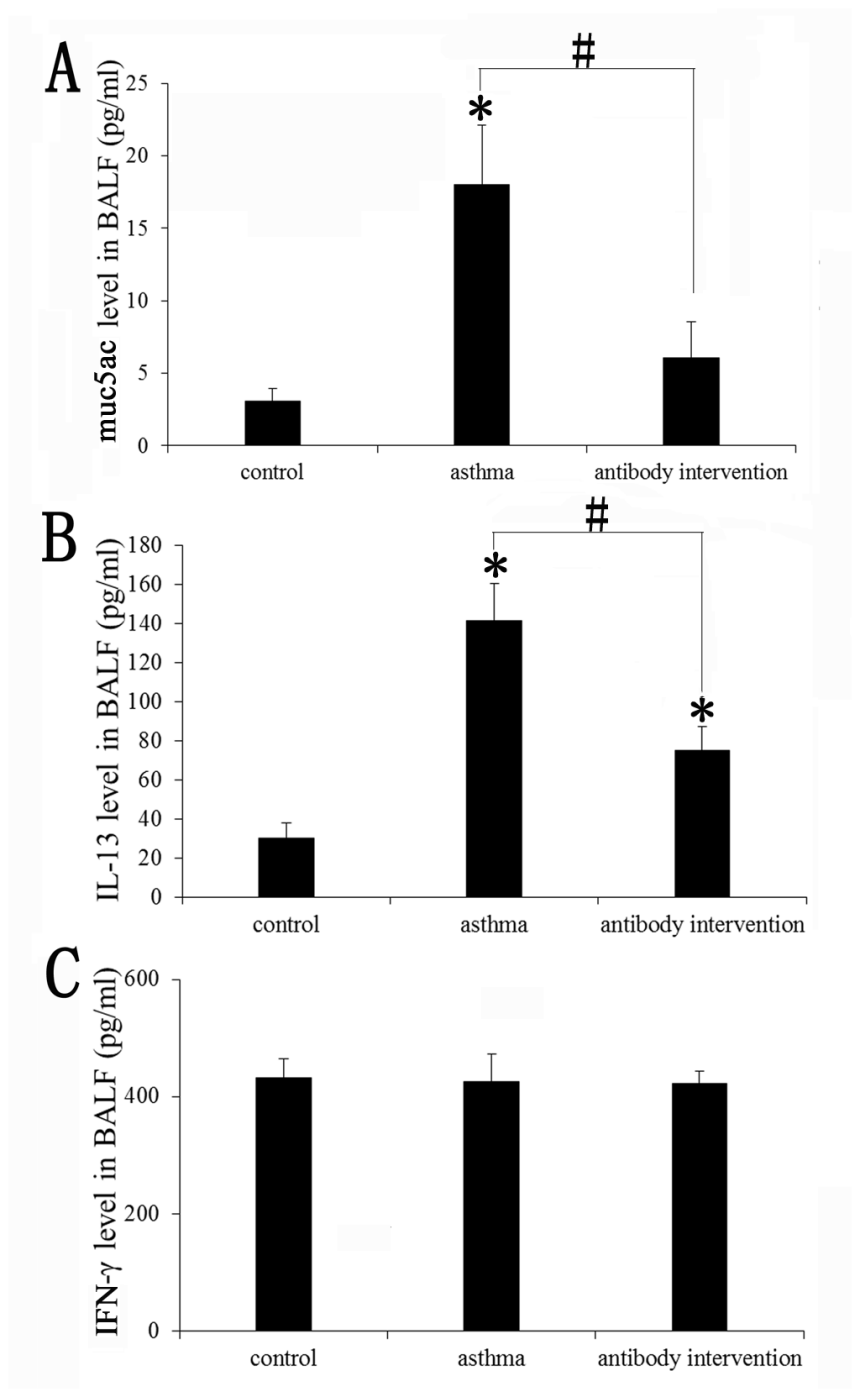
Effects of mCLCA3 antibody on muc5ac and cytokine levels in BALF. BALF was collected 24 h after the last OVA challenge. The muc5ac and cytokine levels were detected by ELISA. (A) The expression of muc5ac in BALF obtained from sensitized mice 24 h after the last PBS aerosol, OVA aerosol and OVA plus antibody intervention challenge. (B) The expression of IL-13 in BALF obtained from sensitized mice 24 h after the last PBS aerosol, OVA aerosol and OVA plus antibody intervention challenge. (C) The expression of IFN-γ in BALF obtained from sensitized mice 24 h after the last PBS aerosol, OVA aerosol and OVA plus antibody intervention challenge. Data are shown as mean± SEM, n = 5 (*P<0.05, compared with the control group; ^#^P<0.05, compared with the asthma group).

There is now clear evidence that muc5ac production is a signature of human and murine asthmatic models. The regulation of muc5ac expression in airway epithelial cells is a critical target for asthma treatment [23]. Evidence suggests that asthma is caused by a Th2 immune response and that some Th2 cytokines, such as IL-13, are involved in the regulation of muc5ac production in asthmatic models [24,25]. Thus, muc5ac, IL-13 and IFN-γ levels in BALF were measured using ELISA. As shown in Figure 2, OVA-challenged mice induced substantial muc5ac and IL-13 expression in BALF compared with the control group. In contrast, IFN-γlevels did not differ between the OVA-challenged group and control group. Treatment with mCLCA3 antibody significantly reduced muc5ac and IL-13 levels in BALF (p<0.05), whereas the antibody had no significant effect on IFN-γ expression. 

### Effect of mCLCA3 antibody on goblet cell hyperplasia and mCLCA3 expression

**Figure 3:**
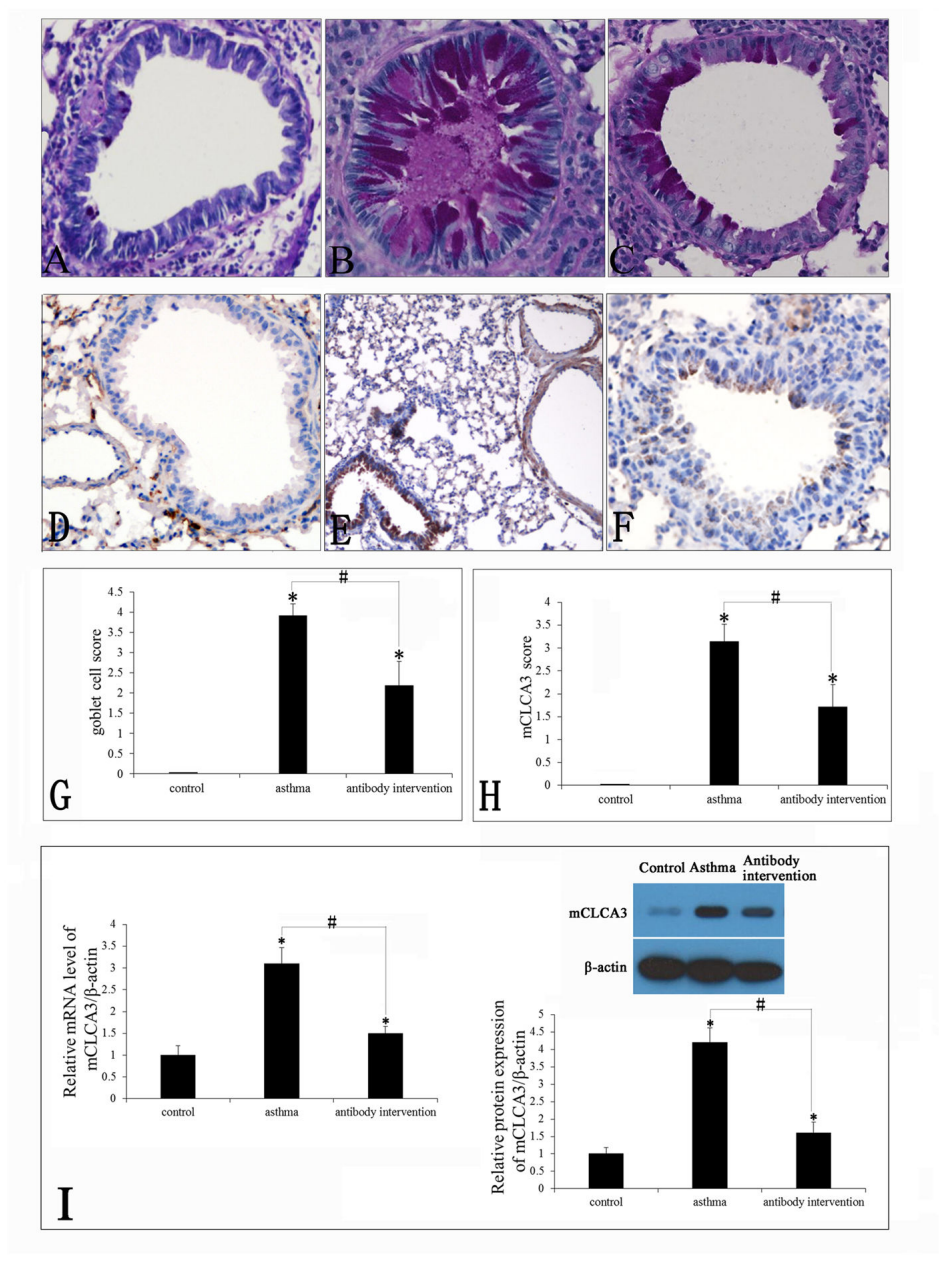
Effects of mCLCA3 antibody on OVA-induced goblet cell hyperplasia and mCLCA3 expression. (A–C) Goblet cell hyperplasia was detected by PAS staining. (A) Control group; (B) Asthma group and (C) mCLCA3 antibody intervention group (magnification x400). (D-F) The expression of mCLCA3 in all groups was detected by immunohistochemistry. (D) Control group; (E) Asthma group and (F) mCLCA3 antibody intervention group (magnification x400). (G) Quantitative analyses of goblet cell hyperplasia in lung sections. (H) Quantitative analyses of mCLCA3 expression in lung sections. (I) The mRNA and protein level of mCLCA3 in PBS aerosol, OVA aerosol and OVA plus antibody intervention group. Data are shown as mean± SEM, n = 5 (*P<0.05, compared with the control group; ^#^P<0.05, compared with the asthma group).

Lung tissue was collected 24 h after the last OVA or PBS aerosol challenge. OVA-induced goblet cell hyperplasia and mucus production were examined by PAS staining. The results showed that there is a marked increase in goblet cell hyperplasia and mucus hypersecretion within the bronchi in the lung in OVA-challenged mice, but not PBS-challenged mice. The mCLCA3 antibody markedly decreased goblet cell hyperplasia (p<0.05) and inhibited mucus hypersecretion. The OVA-induced goblet cell hyperplasia and mucus secretion was significantly abated by mCLCA3 antibody as compared with the control group (Figure 3A–C and G). We next examined the expression of mCLCA3 by immunohistochemistry in all groups of mice. The results suggested that mCLCA3 was not expressed in the goblet cells of normal mice, whereas it was markedly up-regulated in asthmatic mice. In the mCLCA3 antibody intervention asthmatic mouse model, the expression of mCLCA3 was significantly decreased (Figure 3D–F and H). These results indicate that mCLCA3 polyclonalantibody could inhibit goblet cell hyperplasia through the suppression of mCLCA3 expression.

### Effect of mCLCA3 antibody on goblet cell apoptosis

**Figure 4:**
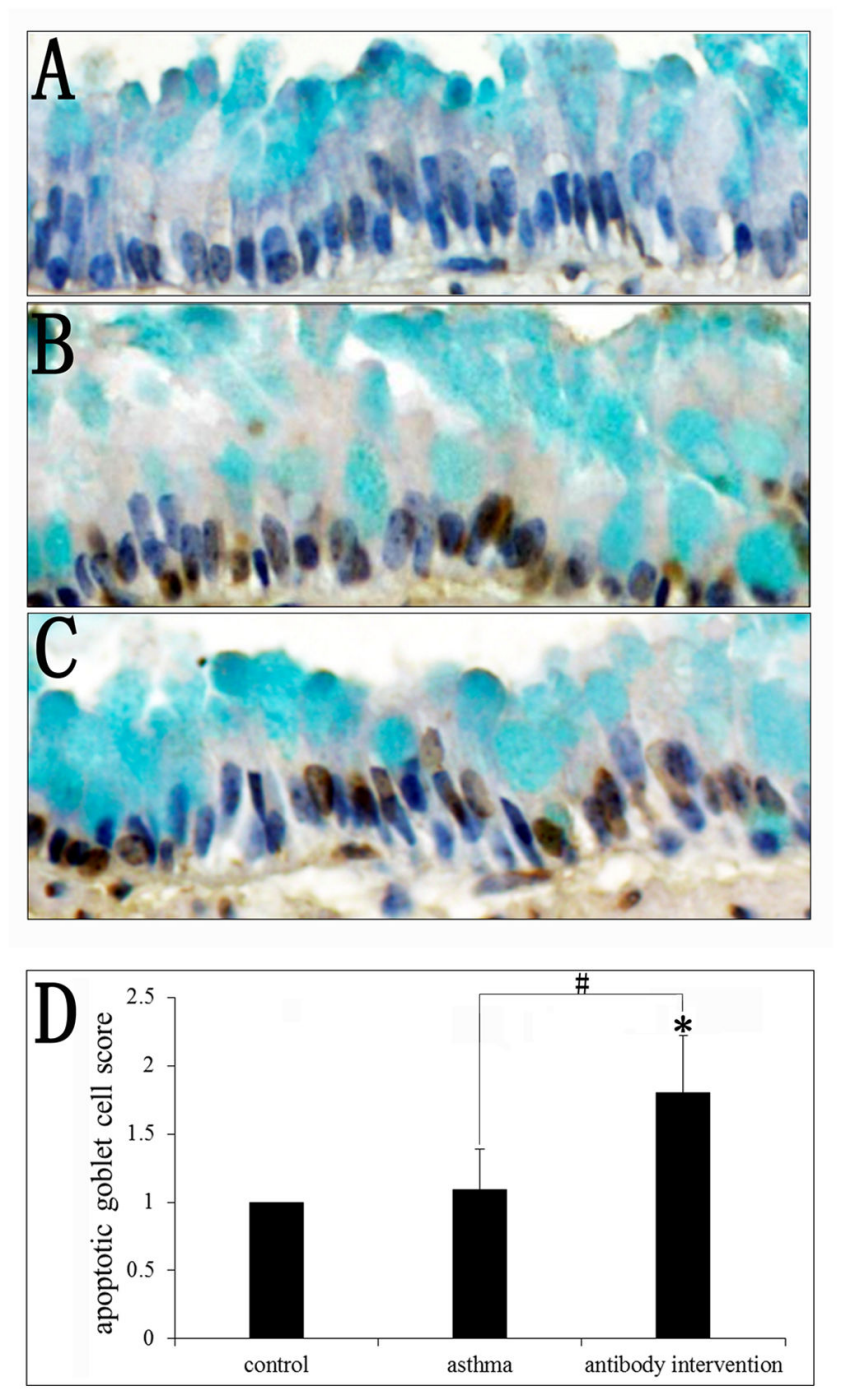
Effects of mCLCA3 antibody on OVA-induced goblet cell apoptosis. (A–C) Cell apoptosis was detected by TUNEL and AB staining. (A) The control group; (B) asthma group and (C) mCLCA3 antibody intervention group (magnification x400). (D) Quantitative analyses of goblet cell apoptosis in lung sections. Data are shown as mean± SEM, n = 5 (*P<0.05, compared with the control group; ^#^P<0.05, compared with the asthma group).

Although the above results indicated that the mCLCA3 antibody could inhibit goblet cell hyperplasia, the mechanism of mCLCA3 antibody action remains unclear. The decrease in goblet cell number may be associated with apoptosis. Thus, we questioned whether there was some connection between the mCLCA3 antibody and goblet cell apoptosis. The apoptosis of goblet cells in all groups of mice was examined using the TUNEL and AB staining method. The results showed that OVA-challenged mice and mCLCA3 antibody intervention mice had higher levels of goblet cell apoptosis compared with the control group, and that mCLCA3 antibody intervention induced a high rate of goblet cell apoptosis (Figure 4).

### Effect of mCLCA3 antibody on apoptosis-associated genes

**Figure 5:**
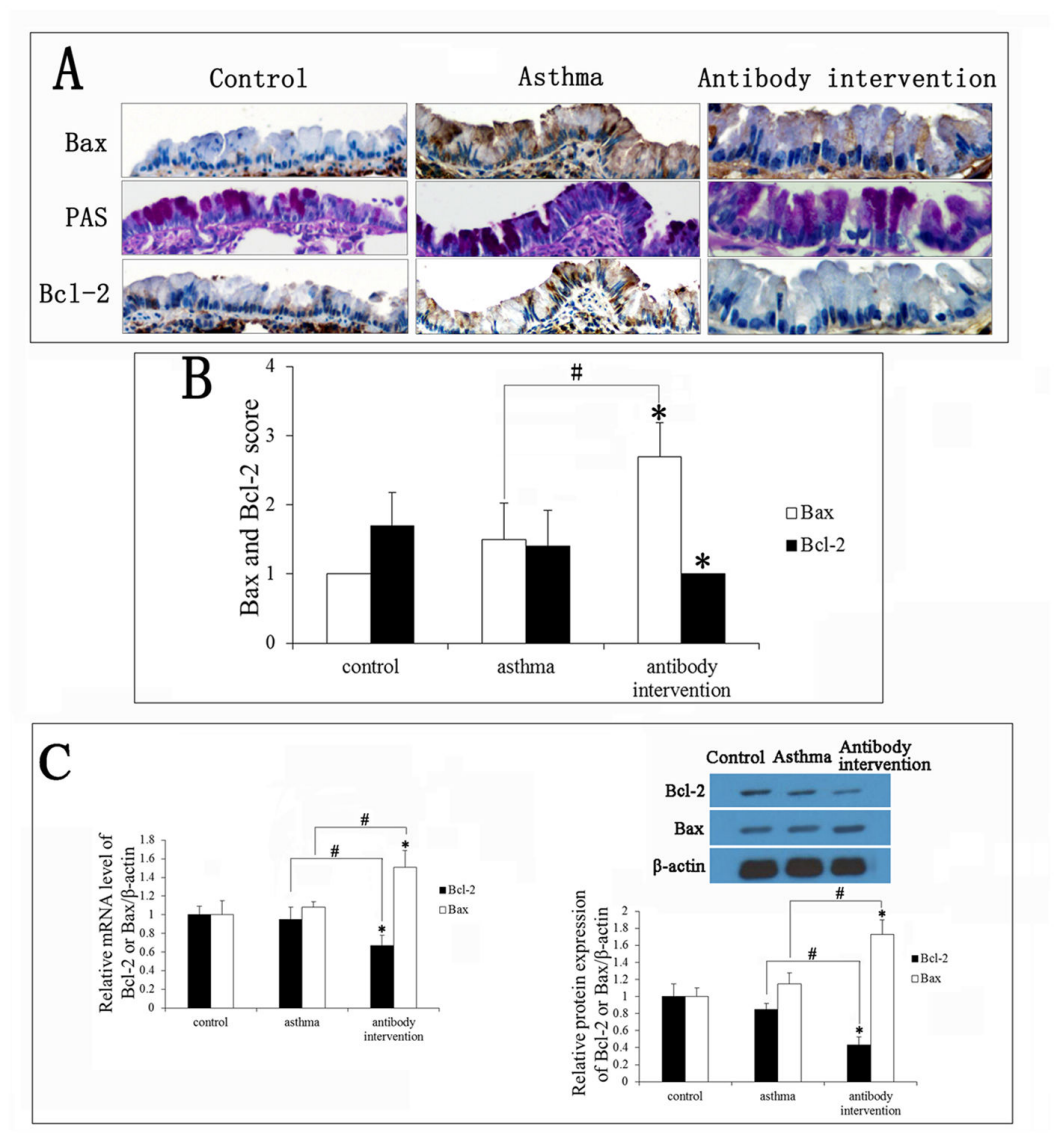
Effects of mCLCA3 antibody on apoptosis-associated genes. (A) Bcl-2 and Bax expression in goblet cells was measured by immunohistochemistry (magnification x400). (B) Quantitative analyses of Bcl-2 and Bax expression levels in goblet cells. (C) The levels of Bcl-2 and Bax mRNA were detected by RT-PCR. (D) The expression of Bcl-2 and Bax in all groups of mice was analyzed by Western blotting. Bcl-2 and Bax expression levels were normalized with β-actin values and are expressed as fold changes compared to control. Data are shown as mean± SEM, n = 5 (*P<0.05, compared with the control group; ^#^P<0.05, compared with the asthma group).

Cell apoptosis appears to be controlled by a series of genes, such as *Bcl-2* and *Bax*. Upregulation of Bcl-2 inhibits multiple forms of cell apoptosis whereas overexpression of Bax accelerates cell apoptosis [26,27]. We next assessed the effect of mCLCA3 antibody on the expression of Bcl-2 and Bax in goblet cells via immunohistochemistry. PAS staining confirmed the location of the goblet cells. A substantial decrease in the Bcl-2 level was observed in the mCLCA3-treated mice, and there was a significant increase in Bax expression in this group compared with the control group (Figure 5A and B). Bcl-2 and Bax mRNA levels were measured by RT-PCR, and protein levels were detected by Western blot and immunohistochemistry. As shown in Figure 5C, down-regulation of the anti-apoptosis gene Bcl-2 was observed in asthmatic mice and antibody intervention mice, whereas the lowest level of Bcl-2 was detected in mCLCA3 antibody intervention mice. In contrast, Bax expression was up-regulated in asthmatic mice and antibody intervention mice, and the highest level of Bax was observed after mCLCA3 antibody treatment. A positive correlation between apoptosis and the ratio of Bax positive cells was indicated by Pearson's correlation coefficient analysis (p<0.05) and there was a negative association between Bcl-2 positive cells and apoptosis (p<0.05).

## Discussion

Asthma is a prevalent disease worldwide, with as many as 300 million people of all ages and ethnic backgrounds suffering from asthma [28]. Allergic asthma is a complex chronic inflammatory disorder characterized by airway inflammation and mucus hypersecretion [29]. The direct link between inflammatory airway disease and *CLCA* expression is particularly interesting; all of these diseases are associated with the overexpression of airway mucus, and there is some evidence that some CLCA proteins are expressed selectively in mucous cells [30–32]. Therefore, the development of mucus hypersecretion in complex airway diseases may be marked or driven by CLCA proteins [10].

The third murine CLCA homologue, mCLCA3 protein, plays an absolutely essential role in allergic asthma. There is growing evidence that mCLCA3 has been identified in goblet cells, and that the suppression of mCLCA3 inhibits goblet cell hyperplasia, whilst overexpression increases goblet cell number in mice [5,6]. CLCA proteins can serve as useful biomarkers as well as significant therapeutic targets for the diagnosis and treatment of patients with chronic inflammatory airway disease [10]. We therefore examined the role of the mCLCA3 antibody in allergic asthma. A well-characterized murine model of OVA-induced allergic asthma was used, in which OVA exposure results in airway inflammation, goblet cell hyperplasia and mucus hypersecretion. Our present findings reveal that mCLCA3 antibody treatment of asthmatic mice effectively ameliorated the symptoms of asthma, such as airway inflammation and muc5ac secretion. The results indicated that the development of airway inflammation and mucus hypersecretion was negatively correlated with the presence of the mCLCA3 antibody. 

There is some evidence that Th2 cells play an important role in the pathogenesis of allergic airway inflammation [33]. Th2 cytokines such as IL-13 and IL-4 are produced by various resident cells such as bronchial epithelial cells, alveolar macrophages, and tissue mast cells as well as infiltrated inflammatory cells such as lymphocytes and eosinophils [19]. There is growing evidence supporting the role of IL-13 in allergic asthma. Our data show that the mCLCA3 antibody significantly reduced the expression of IL-13 both in the BALF and serum. In contrast, the level of IFN-γ, a Th1 cytokine, did not change in the presence of the antibody in BALF. Unfortunately, measurements of IFN-γ in serum were frequently below the level of detection.

Our findings demonstrate a dramatic reduction in mCLCA3 expression with less goblet cell hyperplasia in mCLCA3-treated mice as compared with the control group. In addition, we found that there was a dramatic increase in the rate of goblet cell apoptosis in the asthmatic mouse model. It is well established that the BCL-2 family, comprised of both pro-apoptotic and anti-apoptotic members, has various pairs of antagonist and agonist proteins that regulate programmed cell death (apoptosis) either positively or negatively by as yet unknown mechanisms [34,35]; Bax and Bcl-2 proteins are two members of the Bcl-2 family that have pro-apoptotic and anti-apoptotic effects, respectively. As shown in Figure 5, the expression of Bax was up-regulated in a dose-dependent manner after mCLCA3 antibody treatment. In contrast, the expression of Bcl-2 was significantly down-regulated in mCLCA3 antibody-treated mice. These results suggest that the antibody promots the apoptosis of goblet cells in asthmatic mice by regulating the expression of Bax and Bcl-2. 

Taken together, the findings from our study indicated that the mCLCA3 antibody effectively inhibits goblet cell hyperplasia, airway inflammation and airway mucus hypersecretion. We also observed that the mCLCA3 antibody induced goblet cell apoptosis in mCLCA3 antibody intervention asthmatic mice. The pro-apoptotic effect of the mCLCA3 antibody was associated with Bcl-2 family proteins. The results of our study provide evidence for the first time that the mCLCA3 antibody effectively reduces OVA-induced goblet cell hyperplasia, mCLCA3 expression, airway inflammation, airway mucus hypersecretion and Th2 cytokines in a mouse asthma model. These findings indicate that the mCLCA3 antibody may be an effective agent against human asthma. 

## References

[1] Royce SG, Cheng V, Samuel CS, Tang ML (2012) The regulation of fibrosis in airway remodeling in asthma. Mol Cell Endocrinol 351: 167-175. doi:10.1016/j.mce.2012.01.007. PubMed: 22266540. 22266540

[2] Wong C, Ho C, Ko F, Chan C, Ho A et al. (2001) Proinflammatory cytokines (IL-17, IL-6, IL-18 and IL-12) and Th cytokines (IFN-gamma, IL-4, IL-10 and IL-13) in patients with allergic asthma. Clinical and Experimental Immunology 125: 177-183. 11529906 10.1046/j.1365-2249.2001.01602.x PMC1906135

[3] Hauber H-P, Lavigne F, Hung H-L, Levitt RC, Hamid Q (2010) Effect of Th2 type cytokines on hCLCA1 and mucus expression in cystic fibrosis airways. Journal of Cystic Fibrosis 9: 277-279. doi:10.1016/j.jcf.2010.05.002. PubMed: 20542744. 20542744

[4] Zhou Y, Dong Q, Louahed J, Dragwa C, Savio D et al. (2001) Characterization of a calcium-activated chloride channel as a shared target of Th2 cytokine pathways and its potential involvement in asthma. Am J Respir Cell Mol Biol 25: 486–491. doi:10.1165/ajrcmb.25.4.4578. PubMed: 11694454. 11694454

[5] Komiya T, Tanigawa Y, Hirohashi S (1999) Cloning and identification of the gene gob-5, which is expressed in intestinal goblet cells in mice. Biochem Biophys Res Commun 255: 347-351. doi:10.1006/bbrc.1999.0168. PubMed: 10049711. 10049711

[6] Nakanishi A, Morita S, Iwashita H, Sagiya Y, Ashida Y et al. (2001) Role of gob-5 in mucus overproduction and airway hyperresponsiveness in asthma. Proc Natl Acad Sci U S A 98: 5175-5180. doi:10.1073/pnas.081510898. PubMed: 11296262. 11296262 PMC33183

[7] Vignola AM, Chanez P, Chiappara G, Siena L, Merendino A et al. (1999) Evaluation of apoptosis of eosinophils, macrophages, and T lymphocytes in mucosal biopsy specimens of patients with asthma and chronic bronchitis. J Allergy Clin Immunol 103: 563-573. doi:10.1016/S0091-6749(99)70225-3. PubMed: 10200002. 10200002

[8] Pauli BU, Abdel-Ghany M, Cheng HC, Gruber AD, Archibald HA et al. (2000) Molecular characteristics and functional diversity of CLCA family members. Clin Exp Pharmacol Physiol 27: 901-905. doi:10.1046/j.1440-1681.2000.03358.x. PubMed: 11071307. 11071307

[9] Gaspar KJ, Racette KJ, Gordon JR, Loewen ME, Forsyth GW (2000) Cloning a chloride conductance mediator from the apical membrane of porcine ileal enterocytes. Physiol Genomics 3: 101-111. PubMed: 11015605. 11015605 10.1152/physiolgenomics.2000.3.2.101

[10] Patel AC, Brett TJ, Holtzman MJ (2009) The role of CLCA proteins in inflammatory airway disease. Annu Rev Physiol 71: 425-449. doi:10.1146/annurev.physiol.010908.163253. PubMed: 18954282. 18954282 PMC4017675

[11] Leverkoehne I, Gruber AD (2002) The murine mCLCA3 (alias gob-5) protein is located in the mucin granule membranes of intestinal, respiratory, and uterine goblet cells. J Histochem Cytochem 50: 829-838. PubMed: 12019299. 12019299 10.1177/002215540205000609

[12] Hoshino M, Morita S, Iwashita H, Sagiya Y, Nagi T et al. (2002) Increased expression of the human Ca2+-activated Cl− channel 1 (CaCC1) gene in the asthmatic airway. Am J Respir Crit Care Med 165: 1132-1136. doi:10.1164/ajrccm.165.8.2107068. PubMed: 11956057. 11956057

[13] Toda M, Tulic MK, Levitt RC, Hamid Q (2002) A calcium-activated chloride channel (HCLCA1) is strongly related to IL-9 expression and mucus production in bronchial epithelium of patients with asthma. J Allergy Clin Immunol 109: 246-250. doi:10.1067/mai.2002.121555. PubMed: 11842292. 11842292

[14] Robichaud A, Tuck SA, Kargman S, Tam J, Wong E et al. (2005) Gob-5 is not essential for mucus overproduction in preclinical murine models of allergic asthma. Am J Respir Cell Mol Biol 33: 303–314. doi:10.1165/rcmb.2004-0372OC. PubMed: 15947424. 15947424

[15] Song LQ, Li Y, Li WN, Zhang W, Qi HW et al. (2013) Safety and Immunogenicity of a DNA Vaccine Encoding Human Calcium-Activated Chloride Channel 1 (hCLCA1) in Asthmatic Mice. Int Arch Allergy Immunol 161: 243-251. doi:10.1159/000345972. PubMed: 23548383. 23548383

[16] Mushaben EM, Brandt EB, Hershey GKK, Le Cras TD (2013) Differential effects of rapamycin and dexamethasone in mouse models of established allergic asthma. PLOS ONE 8: e54426. doi:10.1371/journal.pone.0054426. PubMed: 23349887. 23349887 PMC3547928

[17] Le Cras TD, Acciani TH, Mushaben EM, Kramer EL, Pastura PA et al. (2011) Epithelial EGF receptor signaling mediates airway hyperreactivity and remodeling in a mouse model of chronic asthma. Am J Physiol Lung Cell Mol Physiol 300: L414-L421. doi:10.1152/ajplung.00346.2010. PubMed: 21224214. 21224214 PMC3064289

[18] Beilke LD, Aleksunes LM, Olson ER, Besselsen DG, Klaassen CD et al. (2009) Decreased apoptosis during CAR-mediated hepatoprotection against lithocholic acid-induced liver injury in mice. Toxicol Lett 188: 38-44. doi:10.1016/j.toxlet.2009.03.005. PubMed: 19433268. 19433268 PMC2681417

[19] Duan W, Chan JH, Wong CH, Leung BP, Wong WF (2004) Anti-inflammatory effects of mitogen-activated protein kinase kinase inhibitor U0126 in an asthma mouse model. Journal of Immunology 172: 7053-7059. PubMed: 15153527. 10.4049/jimmunol.172.11.7053 15153527

[20] Marino R, Thuraisingam T, Camateros P, Kanagaratham C, Xu YZ et al. (2011) Secretory leukocyte protease inhibitor plays an important role in the regulation of allergic asthma in mice. J Immunol 186: 4433-4442. doi:10.4049/jimmunol.1001539. PubMed: 21335488. 21335488 PMC3104396

[21] Klein E, Weigel J, Buford MC, Holian A, Wells SM (2010) Asymmetric dimethylarginine potentiates lung inflammation in a mouse model of allergic asthma. Am J Physiol Lung Cell Mol Physiol 299: L816-L825. doi:10.1152/ajplung.00188.2010. PubMed: 20889675. 20889675 PMC3006265

[22] Zhang X, Chen W, De Paiva CS, Corrales RM, Volpe EA et al. (2011) Interferon-γ Exacerbates Dry Eye–Induced Apoptosis in Conjunctiva through Dual Apoptotic Pathways. Investigative Ophthalmology and Visual Science 52: 6279-6285. doi:10.1167/iovs.10-7081. 21474767 PMC3176027

[23] Iwashita J, Hongo K, Ito Y, Abe T, Murata J (2013) Regulation of MUC5AC mucin production by the cell attachment dependent pathway involving integrin β1 in NCI-H292 human lung epithelial cells.

[24] Kline JN, Waldschmidt TJ, Businga TR, Lemish JE, Weinstock JV et al. (1998) Cutting edge: modulation of airway inflammation by CpG oligodeoxynucleotides in a murine model of asthma. Journal of Immunology 160: 2555-2559. PubMed: 9510150. 9510150

[25] Kondo M, Tamaoki J, Takeyama K, Nakata J, Nagai A (2002) Interleukin-13 induces goblet cell differentiation in primary cell culture from Guinea pig tracheal epithelium. Am J Respir Cell Mol Biol 27: 536–541. doi:10.1165/rcmb.4682. PubMed: 12397012. 12397012

[26] Dhanabal M, Ramchandran R, Waterman MJ, Lu H, Knebelmann B et al. (1999) Endostatin induces endothelial cell apoptosis. J Biol Chem 274: 11721-11726. doi:10.1074/jbc.274.17.11721. PubMed: 10206987. 10206987

[27] Sentman CL, Shutter JR, Hockenbery D, Kanagawa O, Korsmeyer SJ (1991) bcl-2 inhibits multiple forms of apoptosis but not negative selection in thymocytes. Cell 67: 879-888. doi:10.1016/0092-8674(91)90361-2. PubMed: 1835668. 1835668

[28] Masoli M, Fabian D, Holt S, Beasley R (2004) The global burden of asthma: executive summary of the GINA Dissemination Committee report. Allergy 59: 469-478. doi:10.1111/j.1398-9995.2004.00526.x. PubMed: 15080825. 15080825

[29] Elias JA, Lee CG, Zheng T, Ma B, Homer RJ et al. (2003) New insights into the pathogenesis of asthma. J Clin Invest 111: 291-297. doi:10.1172/JCI200317748. PubMed: 12569150. 12569150 PMC151878

[30] Groneberg DA, Eynott PR, Lim S, Oates T, Wu R et al. (2002) Expression of respiratory mucins in fatal status asthmaticus and mild asthma. Histopathology 40: 367-373. doi:10.1046/j.1365-2559.2002.01378.x. PubMed: 11943022. 11943022

[31] Kuyper LM, Paré PD, Hogg JC, Lambert RK, Ionescu D et al. (2003) Characterization of airway plugging in fatal asthma. Am J Med 115: 6-11. doi:10.1016/S0002-9343(03)00241-9. PubMed: 12867228. 12867228

[32] Hogg JC, Chu F, Utokaparch S, Woods R, Elliott WM et al. (2004) The nature of small-airway obstruction in chronic obstructive pulmonary disease. N Engl J Med 350: 2645-2653. doi:10.1056/NEJMoa032158. PubMed: 15215480. 15215480

[33] Larché M, Robinson DS, Kay AB (2003) The role of T lymphocytes in the pathogenesis of asthma. J Allergy Clin Immunol 111: 450-463. doi:10.1067/mai.2003.169. PubMed: 12642820. 12642820

[34] Knudson CM, Korsmeyer SJ (1997) Bcl-2 and Bax function independently to regulate cell death. Nat Genet 16: 358-363. doi:10.1038/ng0897-358. PubMed: 9241272. 9241272

[35] Antonsson B, Conti F, Ciavatta A, Montessuit S, Lewis S et al. (1997) Inhibition of Bax channel-forming activity by Bcl-2. Science 277: 370-372. doi:10.1126/science.277.5324.370. PubMed: 9219694. 9219694

